# Supplementary material for: Natural Variation in Diauxic Shift between Patagonian Saccharomyces eubayanus Strains
Source: mSystems. 2022 Dec 5;7(6):e00640-22. doi: 10.1128/msystems.00640-22 (PMC9765239; doi:10.1128/msystems.00640-22)
Supplement: FIG S1 [file msystems.00640-22-s0007.pdf]

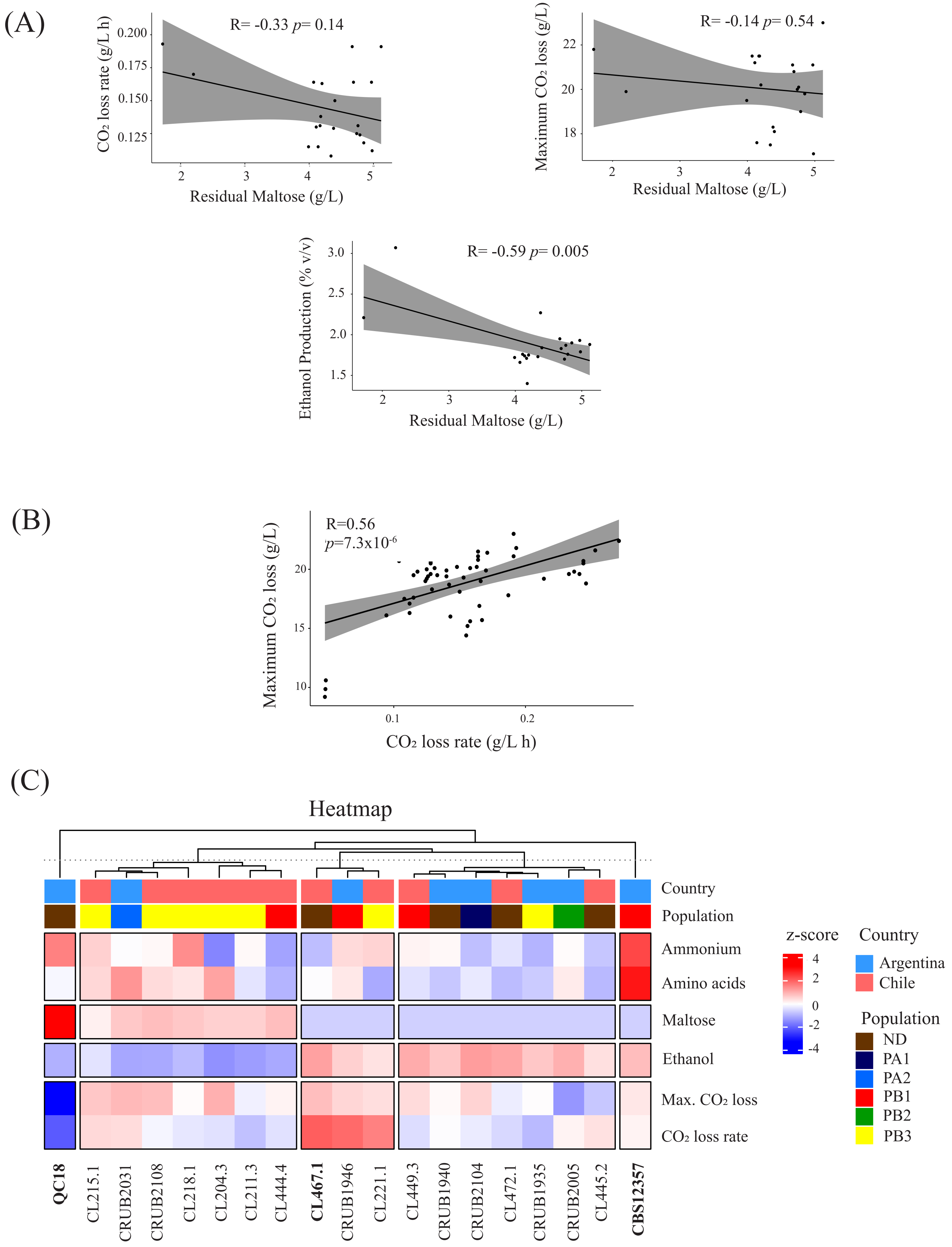

Figure S1. Pearson correlation for residual maltose versus CO_2 loss rate, maximum CO_2 loss and ethanol production. (**B**) Pearson correlation for CO_2 loss rate versus Maximum CO_2 loss. (**C**) Hierarchically clustered heatmap of kinetic parameters. Phenotypic values are calculated as normalized z-scores.
