# Supplementary material for: Natural Variation in Diauxic Shift between Patagonian Saccharomyces eubayanus Strains
Source: mSystems. 2022 Dec 5;7(6):e00640-22. doi: 10.1128/msystems.00640-22 (PMC9765239; doi:10.1128/msystems.00640-22)
Supplement: FIG S2 [file msystems.00640-22-s0008.pdf]

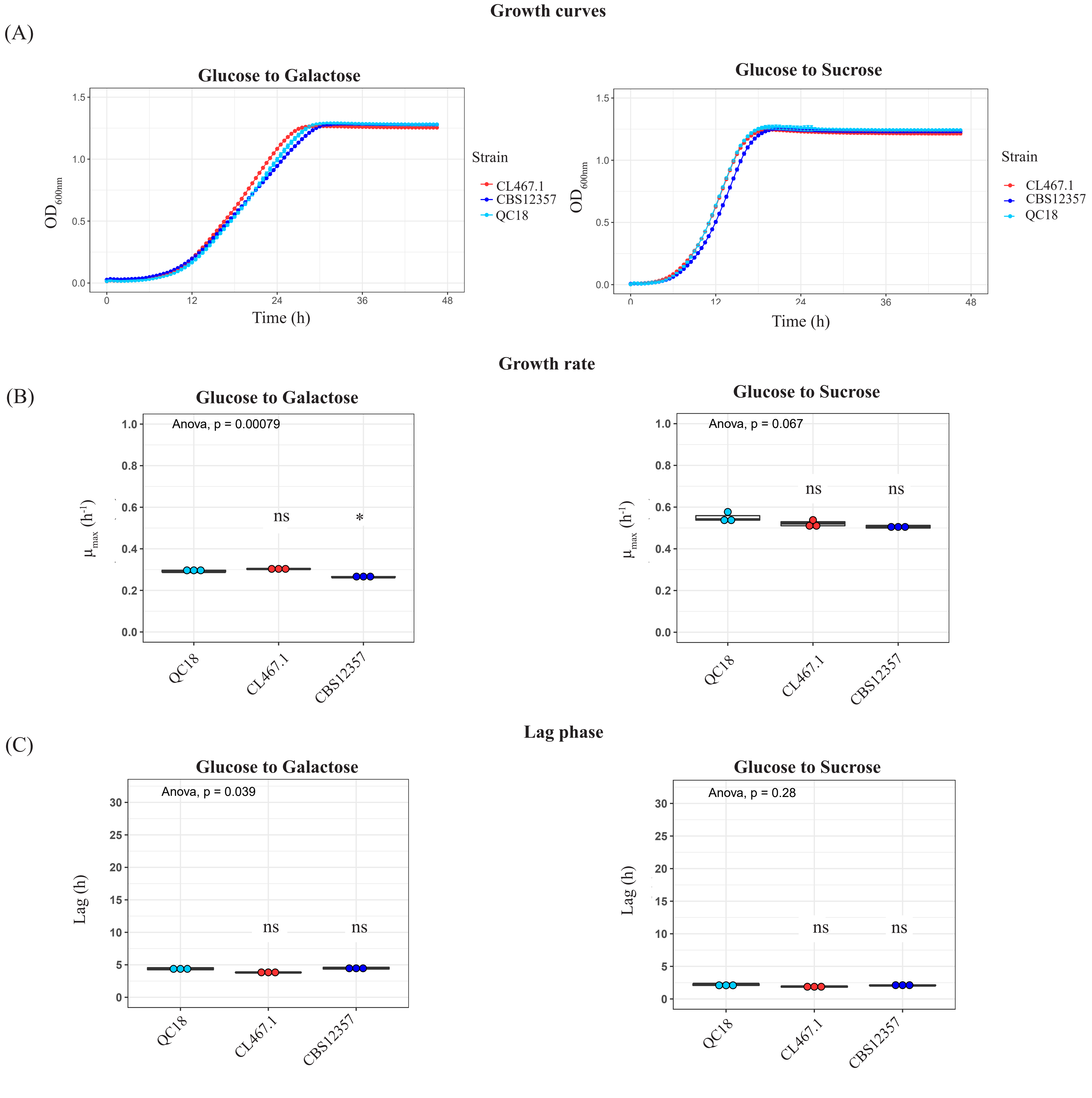

Figure S2. (**A**) Growth curves obtained after glucose-galactose and glucose-sucrose shift. (**B**) Growth rates and (**C**) Lag times after glucose-galactose and glucose-sucrose shift.
