# Supplementary material for: Natural Variation in Diauxic Shift between Patagonian Saccharomyces eubayanus Strains
Source: mSystems. 2022 Dec 5;7(6):e00640-22. doi: 10.1128/msystems.00640-22 (PMC9765239; doi:10.1128/msystems.00640-22)
Supplement: FIG S3 [file msystems.00640-22-s0009.pdf]

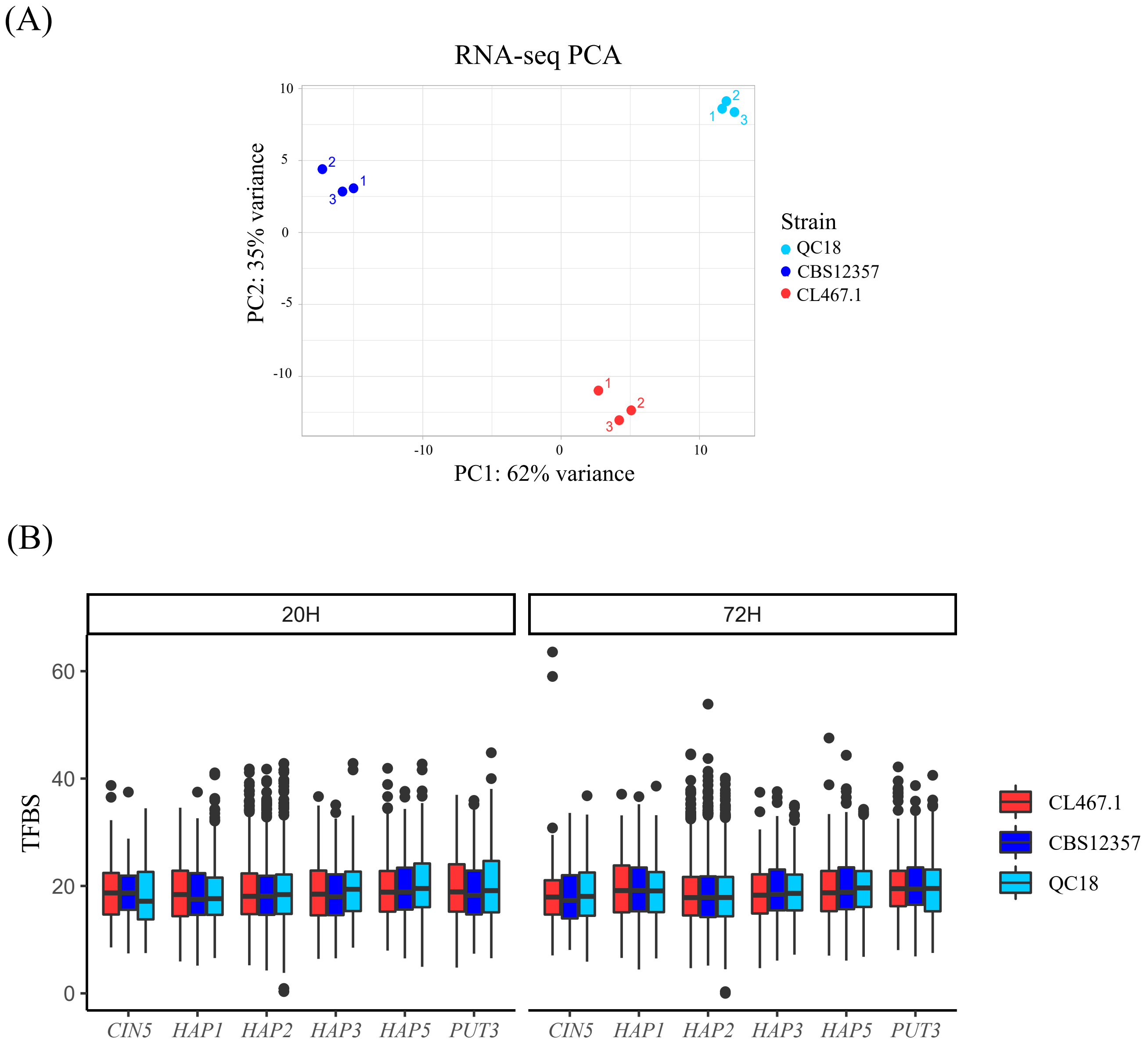

Figure S3. (A) Principal components analysis of DEGs. (B) Transcription factor binding scores (TFBS) for Cin5p, Hap1, Hap2, Hap3, Hap5 and Put3 at 20 h and 72 h.
