# Supplementary material for: Natural Variation in Diauxic Shift between Patagonian Saccharomyces eubayanus Strains
Source: mSystems. 2022 Dec 5;7(6):e00640-22. doi: 10.1128/msystems.00640-22 (PMC9765239; doi:10.1128/msystems.00640-22)
Supplement: FIG S4 [file msystems.00640-22-s0010.pdf]

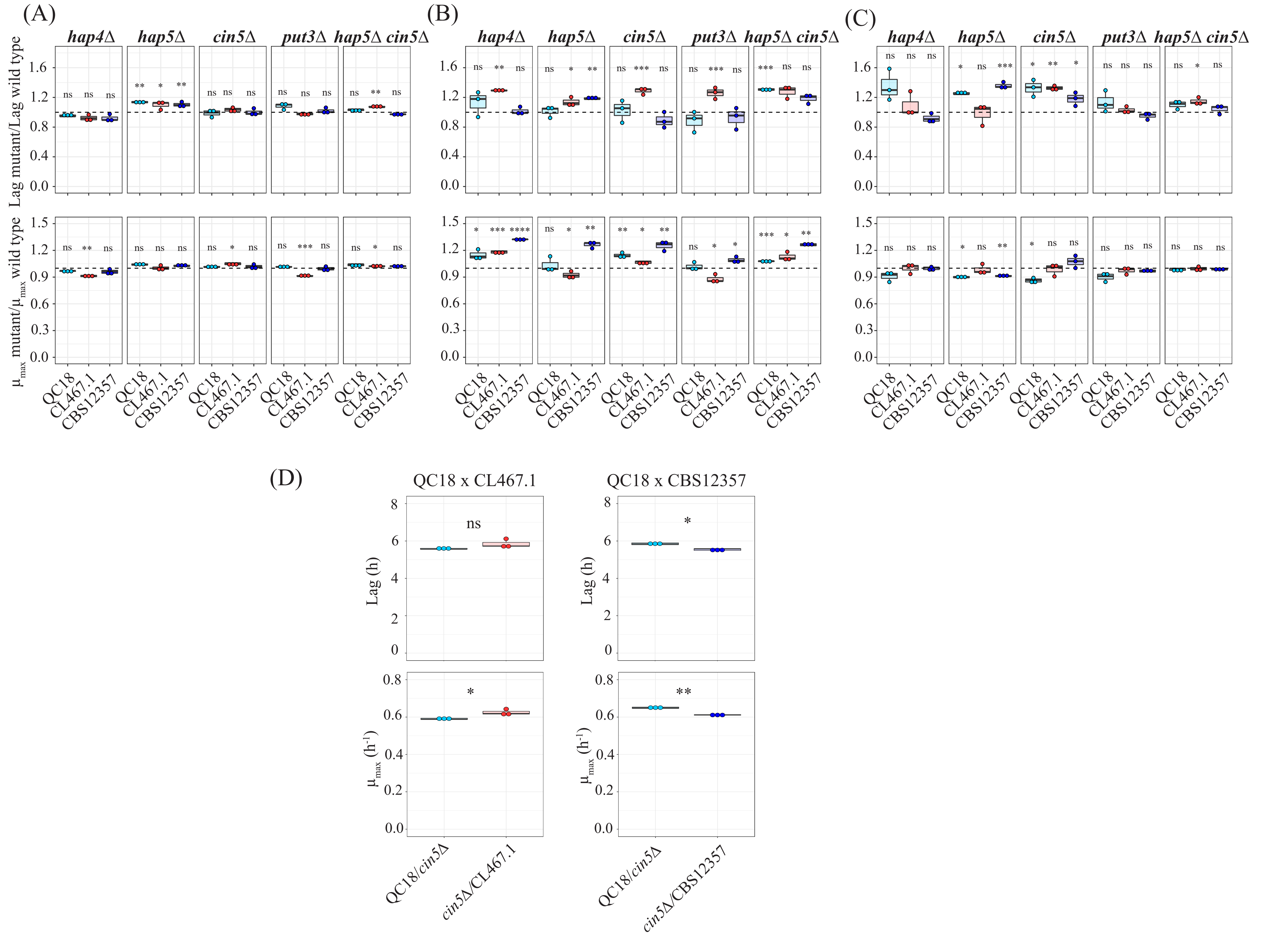

Figure S4. Effect of Hap4p, Hap5, Cin5p and Put4p on the growth in glucose (A), after glucose-galactose shift (B) and after glucose-sucrose shift (C). Reciprocal hemygosity analysis for *CIN5* gene on kinetic parameters after glucose-maltose shift in QC18 x CL467.1 and QC18 x CBS12357 strains (D).
